# Hidden taxonomic and taphonomic diversity revealed by mayflies (Ephemeroptera: Hexagenitidae) from mid-Cretaceous Kachin amber

**DOI:** 10.1038/s41598-026-46621-8

**Published:** 2026-04-27

**Authors:** Nicolas Adrian Stagg, Hui Jiang, Frank Tomaschek, Arnold H. Staniczek, Bastian Mähler, Jana Bruthansová, Thet Tin Nyunt, Roman J. Godunko

**Affiliations:** 1 Institute for Geology and Palaeontology, University of Münster, 48149 Münster, Germany; 2 Department for Paleontology, Bonn Institute for Organismic Biology (BIOB), University of Bonn, 53115 Bonn, Germany; 3 Senckenberg Forschungsstation Grube Messel, Senckenberg Forschungsinstitut und Naturmuseum Frankfurt/M, 64409 Messel, Germany; 4 Institute of Geology and Paleontology, Charles University, 12843 Prague, Czech Republic; 5 State Key Laboratory of Paleobiology and Stratigraphy, Nanjing Institute of Geology and Palaeontology, Chinese Academy of Sciences, Nanjing, 210008 China; 6 Institute of Geosciences, University of Bonn, 53115 Bonn, Germany; 7 Department of Entomology, State Museum of Natural History Stuttgart, Rosenstein 1, 70191 Stuttgart, Germany; 8 Department of Palaeontology, National Museum, Cirkusová 1740, 19300 Prague 9, Czech Republic; 9 Department of Geological Survey and Mineral Exploration, Ministry of Natural Resources and Environmental Conservation, Myanmar Gems Museum, 15011 Nay Pyi Taw, Myanmar; 10 Biology Centre of the Czech Academy of Sciences, Institute of Entomology, Branišovská 31, 37005 České Budějovice, Czech Republic; 11 Department of Invertebrate Zoology and Hydrobiology, Faculty of Biology and Environmental Protection, University of Lodz, Banacha 12/16, 90237 Łódź, Poland; 12 State Museum of Natural History, National Academy of Sciences of Ukraine, Teatralna 18, Lviv, 79008 Ukraine

**Keywords:** Palaeoecology, Palaeontology, Mineralogy

## Abstract

Hexagenitidae is an extinct family of Ephemeroptera with a fossil record spanning from the Early Jurassic to the Early Cretaceous. However, in mid-Cretaceous Kachin amber, one of the most diverse insect-bearing biotas known to date, only a single species, *Hexameropsis elongatus* Lin et al., has been described, and it is known solely from male adults. In this study, we describe three well-preserved species of Hexagenitidae from Kachin amber, including one new genus and species based on a female imago, *Crehkahtihtengia dongi* gen. et sp. nov., a new species, *Hexameropsis fehrmannorum* sp. nov., and a putative representative of *Hexameropsis* Tshernova & Sinitshenkova, highlighting that the diversity of this family in Kachin amber is greater than previously recognized. Ecologically, Hexagenitidae in Burmese amber likely represents rhithral mayflies associated with running-waters habitats, in contrast to the predominantly lacustrine members of this family known from Jurassic and Cretaceous compression fossils. We conducted a taphonomic analysis of the new mayfly specimens using light microscopy, fluorescence microscopy, scanning electron microscopy (SEM), energy-dispersive and wavelength-dispersive X-ray spectroscopy (EDS and WDX), X-ray micro-computed tomography (Micro-CT), and Raman spectroscopy. Our study provides evidence that clay minerals were involved in the structural preservation of insect fossils in amber. We found the co-preservation of calcite, quartz, pyrite, and hydrous phyllosilicates (likely chamosite/berthierine or other clay minerals) within each individual fossil specimen, in which these phases infilled internal body voids and likely helped to maintain body shapes. This multi-phase assemblage indicates a complex, multi-stage depositional history within a single inclusion and highlights the diversity of mineralization-based preservation pathways in Kachin amber. Together with previous work on the taphonomy of Kachin amber inclusions, these results suggest that mineralization products associated with preservation of biological structures in amber may reflect evolving fluid compositions, fluctuating redox conditions, and dynamic transformations of pore space within the amber and at its interface with the surrounding environment.

## Introduction

Ephemeroptera (mayflies) are characterized by an amphibiotic life cycle, with entirely aquatic nymphs and two short-lived winged stages on land (subimago and imago), which closely links them to evolutionary processes and environmental changes in lakes and rivers and, through their critical role in the food web, makes them vital prey for a wide range of predators and important contributors to ecosystem nutrient cycling^1–4^. Extant mayflies represent one of the less speciose insect orders, comprising over 3,330 species in 42 families, and are distributed nearly worldwide, except for Antarctica and several oceanic islands^1,2^. Phylogenetically, mayflies are among the earliest pterygote insect lineages and are placed within Hydropalaeoptera^5^, with a stem-group traceable back to the Late Carboniferous^6,7^. Sroka et al. (2023)^3^ compiled a comprehensive list of all described fossil mayfly occurrences identifiable to at least the genus level, totalling 255 records from 140 genera.

Among these fossil records, recent discoveries from mid-Cretaceous Kachin amber, which forms an important component of the broader Burmese amber record,  have provided exceptional insights into the diversity and early evolution of Mesozoic Ephemeroptera^8^. At the time of submission, 12 genera and 16 species of fossil mayflies (Ephemeroptera) had been reported from Burmese amber (Table 1), and a total of 11 families had been mentioned: Australiphemeridae McCafferty, 1991^95^, Baetidae Leach, 1851^96^, Ephemeridae Latreille, 1810^12^, Heptageniidae Needham, 1901^13^, Isonychiidae Burks, 1953^97^, Leptophlebiidae Banks, 1990 ^98^, Oculephemeridae Zheng and Chen, 2024^15^, Prosopistomatidae Laméere, 1917^99^, Siphlonephemerellidae Chen and Zheng, 2023^93^, Vietnamellidae Allen, 1984^17^, and Hexagenitidae Laméere, 1917^18^, the latter family being the focus of this contribution. By the time of publication, seven additional papers on mayflies from Burmese amber had been published^92,^ ^100–105^but these are not discussed further here as they fall outside the scope of the present study.

**Table 1:** The fossil records of Ephemeroptera from Burmese amber (as of October 2025).

Family	Genera	Species	References
Australiphemeridae	*Crepotamanthus*	*Crepotamanthus spinitarsus* Zheng & Chen, 2023	Zheng & Chen, 2023^19^
	*Nanophemera*	*Nanophemera myanmarensis* McCafferty & Santiago-Blay, 2008	McCafferty & Santiago-Blay, 2008^10^
Baetidae	*Myanmarella*	*Myanmarella rossi* Sinitshenkova, 2000	Sinitshenkova, 2000^106^
	*Vetuformosa*	*Vetuformosa buckleyi* Poinar, 2011	Poinar, 2011^11^
Ephemeridae			Ross, 2024^ 8^
Heptageniidae		Ecdyonurinae gen. et sp. indet.	Hrivniak et al., 2020^21^
	*Longiheptagenia*	*Longiheptagenia bipartita* Yang, Zhao & Ren, 2023	Yang et al., 2023^22^
		*Longiheptagenia elegantula* Yang, Zhao & Ren, 2023	Yang et al., 2023^22^
Hexagenitidae	*Hexameropsis*	*Hexameropsis elongatus* Lin, Shih & Ren, 2018	Lin et al., 2018^23^
		*Hexameropsis cf. elongatus*	This study
		*Hexameropsis fehrmannorum* sp. nov.	This study
	*Crehkahtihtengia* gen. nov.	*Crehkahtihtengia dongi* gen. et sp. nov.	This study
Isonychiidae	*Fujiwaranychia*	*Fujiwaranychia chiyokoae* Chen & Zheng, 2025	Chen & Zheng, 2025^92^
Leptophlebiidae	*Kachinophlebia*	*Kachinophlebia zhouchangfai* Chen & Zheng, 2022	Chen & Zheng, 2022^14^
	*Crephlebia*	*Crephlebia zhoui* Chen & Zheng, 2024	Chen & Zheng, 2024^24^
Oculephemeridae	*Oculephemera*	*Oculephemera mazhenxingi* Zheng & Chen, 2023	Zheng & Chen, 2023^15^
Prosopistomatidae	*Proximicorneus*	*Proximicorneus rectivenius* Lin, Shih & Ren, 2018	Lin et al., 2018^16^
Siphlonephemerellidae	*Siphlonephemerella*	*Siphlonephemerella mupengxui* Chen & Zheng, 2023	Chen & Zheng, 2023^ 93^
Vietnamellidae	*Burmella* = *Burmaheptagenia*	*Burmella paucivenosa* Godunko, Martynov & Staniczek, 2021	Godunko et al., 2021^25^
		*Burmella clypeata* Godunko, Martynov & Staniczek, 2021	Godunko et al., 2021^25^
		*Burmella zhouchangfai* Chen & Zheng, 2023	Chen & Zheng, 2023^19^; Godunko et al., 2025^9^
		*Burmella inconspicua* Godunko & Staniczek, 2025	Godunko et al., 2025^9^

The family Hexagenitidae in Burmese amber was previously known only from a single genus and species, *Hexameropsis elongatus* Lin et al., 2018^8,23^. The fossil record of Hexagenitidae spans from the Lower Jurassic to the Lower Cretaceous^3,16,23,26^ and includes 14 genera and 26 species (see Table 2), as well as a species *incertae sedis*, among these, 7 genera and 12 species are identified from winged stage. The distinctive feature of the imaginal stages is the forewing venation, characterized by a branched CuA in the forewing and multiple successive loop-like triads occurring between CuA_1_ and CuA_2_^27^. So far, the earliest fossil record of Hexagenitidae are from the Middle Jurassic of western Mongolia^28^ and northern China^29^, indicating the presence of this family in eastern Eurasia by that time. By the Late Jurassic, Hexagenitidae had expanded their range, with fossils reported from the Transbaikalia region of Russia^28^, southwestern Mongolia^30^, and the Solnhofen Limestone of Germany^31^, indicating a widespread distribution across Eurasia by that time. From the Early Cretaceous to the earliest Late Cretaceous, Hexagenitidae are known not only in Eurasia^30–35^, but also from Canada^26^, Brazil^36^, Algeria^37^, and Myanmar^23^.

**Table 2:** The fossil records of Hexagenitidae.

Genus	Species	Age (Ma)	Geological age (epoch/stage)	Life stage	Locality	References
*Hexagenites*	*Hexagenites weyenberghi*	151–145	Late Jurassic, Tithonian	Imago	Solnhofen, Germany	Scudder, 1880^38^; Willmann, 2007^31^
	*Hexagenites cellulosus*	151–145	Late Jurassic, Tithonian	Imago	Solnhofen, Germany	Hagen, 1862^39^; Willmann, 2007^31^
*Ephemeropsis*	*Ephemeropsis trisetalis*	126–113	Early Cretaceous, Barremian-Aptian	Nymph & imago	Transbaikalia, Russia, Mongolia	Eichwald, 1864^40^; Huang et al., 2007^33^
	*Ephemeropsis martynovae*	125–113	Early Cretaceous, Aptian	Imago	Transbaikalia, Russia	Tshernova, 1961^41^; Willmann, 2007^31^
*Hexameropsis*	*Hexameropsis selini*	125–113	Early Cretaceous, Aptian	Imago	Ukraine	Tshernova & Sinitshenkova, 1974^41^; Willmann, 2007^31^
	*Hexameropsis elongatus*	99	Late Cretaceous, Cenomanian	Imago	Kachin, Myanmar	Lin et al. 2018^23^
	*Hexameropsis cf. elongatus*	99	Late Cretaceous, Cenomanian	Imago	Kachin, Myanmar	This study
	*Hexameropsis fehrmannorum sp. nov.*	99	Late Cretaceous, Cenomanian	Imago	Kachin, Myanmar	This study
*Crehkahtihtengia*gen. nov.	*Crehkahtihtengia dongi* gen. et sp. nov.	99	Late Cretaceous, Cenomanian	Imago	Kachin, Myanmar	This study
*Mongologenites*	*Mongologenites laqueatus*	125–113	Early Cretaceous, Aptian	Nymph & imago	Mongolia	Sinitshenkova, 1986^42^; Willmann, 2007^31^
*Epicharmeropsis*	*Epicharmeropsis quadrivenulosus*	126–124	Early Cretaceous, Earliest Aptian	Nymph & imago	China	Huang et al., 2007^33^
	*Epicharmeropsis hexavenulosus*	126–124	Early Cretaceous, Earliest Aptian	Nymph & imago	Hebei, China	Huang et al., 2007^33^
*Protoligoneuria*	*Protoligoneuria limai*	115–113	Early Cretaceous, Latest Aptian	Nymph & imago	Southern Ceará state, Brazil	Demoulin, 1955^43^; Storari et al., 2022^36^
	*Protoligoneuria heloisae*	115–113	Early Cretaceous, Latest Aptian	Imago	Southern Ceará state, Brazil	Storari et al., 2022^36^
*Incertae sedis*	Hexagenitidae *incertae sedis*	115–113	Early Cretaceous, Latest Aptian	Imago	Southern Ceará state, Brazil	Storari et al., 2022^36^
*Costalimella*	*Costalimella zucchii*	115–113	Early Cretaceous, Latest Aptian	Imago	Southern Ceará state, Brazil	Zamboni, 2001^44^; Brandão et al., 2021^45^
*Cratohexagenites*	*Cratohexagenites longicercus*	115–113	Early Cretaceous, Latest Aptian	Nymph & imago	Southern Ceará state, Brazil	Staniczek, 2007^46^; Storari et al., 2022^36^
	*Cratohexagenites minor*	115–113	Early Cretaceous, Latest Aptian	Nymph	Southern Ceará state, Brazil	Staniczek, 2007^46^; Storari et al., 2022^36^
*Baikalogenites*	*Baikalogenites firmus*	115–113	Early Cretaceous, Aptian	Nymph	Khasurty, Western Transbaikalia, Russia	Sinitshenkova, 2017^32^
*Caenoephemera*	*Caenoephemera shangyuanensis*	126–123	Early Cretaceous, Earliest Aptian	Nymph	Changbodianzi, Shangyuan, Jianshangou. Loaoning, China	Lin & Huang, 2001^47^; Willmann, 2007^31^
*Hexameropsis*	*Hexameropsis africana*	133–126	Early Cretaceous, Hauterivian	Nymph	Algeria	Sinitshenkova, 1975^34^; Vršanský et al., 2021^37^
*Siberiogenites*	*Siberiogenites branchicillus*	126–123	Early Cretaceous, Latest Aptian	Nymph	Huangbanjigou, Chaomidian Village, China	Huang et al., 2011^34^
	*Siberiogenites medius*	131–126	Early Cretaceous, Hauterivian	Nymph	Mongolia	Shcherbakov, 1989^35^
	*Siberiogenites recticostalis*	126–121	Early Cretaceous, Barremian	Nymph	Chernovskie Kopi outcrop 9, Russia	Sinitshenkova, 2000^20^
	*Siberiogenites mongolicus*	149–143	Late Jurassic, Tithonian	Nymph	Shar-Teg, Mongolia	Sinitshenkova, 2002^30^
	*Siberiogenites rotundatus*	165–161	Middle Jurassic, Callovian	Nymph	Mongolia	Sinitshenkova, 1985^28^
	*Siberiogenites angustatus*	161–155	Late Jurassic, Oxfordian	Nymph	Transbaikalia, Russia	Sinitshenkova, 1985^28^
*Shantous*	*Shantous lacustris*	167–164	Middle Jurassic, Callovian	Nymph	Ningcheng, Inner Mongolia, China	Zhang & Kluge, 2007^29^
*Protoligoneuria*	*Protoligoneuria borealis*	99.7–94.3	Late Cretaceous, Cenomanian	Nymph	Labrador, Canada	Mueller & Demers-Potvin, 2024^26^

In this study, we report a new genus, two new species, and a specimen tentatively assigned to a previously described hexagenitid species based on new material from mid-Cretaceous amber from Kachin state, northern Myanmar (~ 99 Ma), and we detail their morphological characteristics and comparing them with known adult taxa within the family. These findings expand the fossil records of Hexagenitidae in Kachin amber and, more broadly, enhance our understanding of Cretaceous Ephemeroptera. The new taxa reveal markedly greater morphological and species diversity within Hexagenitidae than previously recognized, suggesting that the taxonomic richness of the group in this depositional context has been underestimated.

In addition to their taxonomic significance, fossils also provide important insights into taphonomy, the factors and processes that shape preservation. Taphonomic features show how organism-environment interactions during burial and diagenesis influence the retention of morphological and molecular signals, reveal preservation biases, and clarify pathways such as mineralization and the preservation of soft-tissue structures, thereby informing reconstruction of past biological history and environmental conditions^48–53^. To this end, in addition to the taxonomic analysis, we investigated the preservation features of the newly discovered mayfly fossils using scanning electron microscopy (SEM), energy-dispersive and wavelength-dispersive X-ray spectroscopy (EDS and WDX), X-ray micro-computed tomography (Micro-CT), and Raman spectroscopy on exposed surfaces of the specimens.

As these fossils are preserved in amber, this depositional context provides a rare and unique opportunity to explore taphonomic processes that can be compared with those observed in rock-hosted fossils. Early studies commonly interpreted amber inclusions as mummified remains or hollow molds, occasionally accompanied by carbonaceous coatings or fillings^51,54–61^. However, several studies have reported mineralization in amber inclusions—such as pyritization, transformation of bioapatite to fluorapatite, calcification and silicification^60,62–68^—indicating that biological inclusions may undergo complex post-entombment modification, such as mineral replacement, degradation, and recrystallization, yet systematic taphonomic studies of amber fossils remain limited.

Jiang et al.^68^ conducted an initial systematic taphonomic study of Kachin amber inclusions, focusing on mineralized preservation. Their results indicate that taphonomic pathways in the diverse insect groups examined are not taxon-dependent. In Burmese amber, preservation commonly involves calcification and silicification and may be accompanied by minor amounts of carbonaceous material, pyrite, iron oxides, and phyllosilicate minerals. Their study of mineralized inclusions underscores the role of diagenesis and fluids (e.g. sediment pore water, diagenetic fluid and groundwater) at different burial stages in shaping the final preservation of amber fossils. Such taphonomic processes affect not only the degree of morphological fidelity but may also influence interpretations of the biological and ecological traits of the preserved organisms.

In our study, we found that the specimens preserve mineralized infills composed of multiple co-occurring mineral phases, rather than a single mineral phase such as quartz or calcite, further highlighting the diversity of mineralization-based fossil preservation in Kachin amber. The presence of multiple mineral phases within a single specimen suggests that individual inclusions may have experienced multiple episodes of post-entombment alteration involving chemically distinct fluids. These observations further support for the conclusions for Jiang et al. (2022)^68^, and reinforce the view that Kachin amber does not always behave as a closed system. Instead, fluids of varying origins and ages may have episodically infiltrated the resin/amber matrix through microcracks, and ultimately influenced the taphonomic quality of the enclosed fossils. Our observations underscore the taphonomic complexity and variability of mineralization pathways in amber. The preservation patterns documented in this study expand our understanding of diagenetic processes in Burmese amber and point to a more dynamic and heterogeneous post-entombment environment than previously recognized.

## Results

### Systematic palaeontology and taphonomic preservation

**Ephemeroptera Latreille**,** 1810**^12^.

**Hexagenitidae Lameere**,** 1917**^18^.

Crehkahtihtengia dongi Stagg, Jiang, Staniczek & Godunko gen. et sp. nov. LSID urn: lsid: zoobank.org: pub: E14F638C-8A06-4D25-8577-C6CC0DF2B825.

Figures 1, 2 and 3

**Type species**. *Crehkahtihtengia dongi***gen. et sp. nov.**

**Species composition.** Monospecific.

**Etymology.** The generic name *Crehkahtihtengia* is formed by combining the prefix Cre-, referring to the Cretaceous, with hkahti-hteng, a Jinghpaw (Jingpho/Kachin) term for “mayfly”, and the Latin suffix -ia. The gender of the generic name is feminine.

**Fig. 1:**
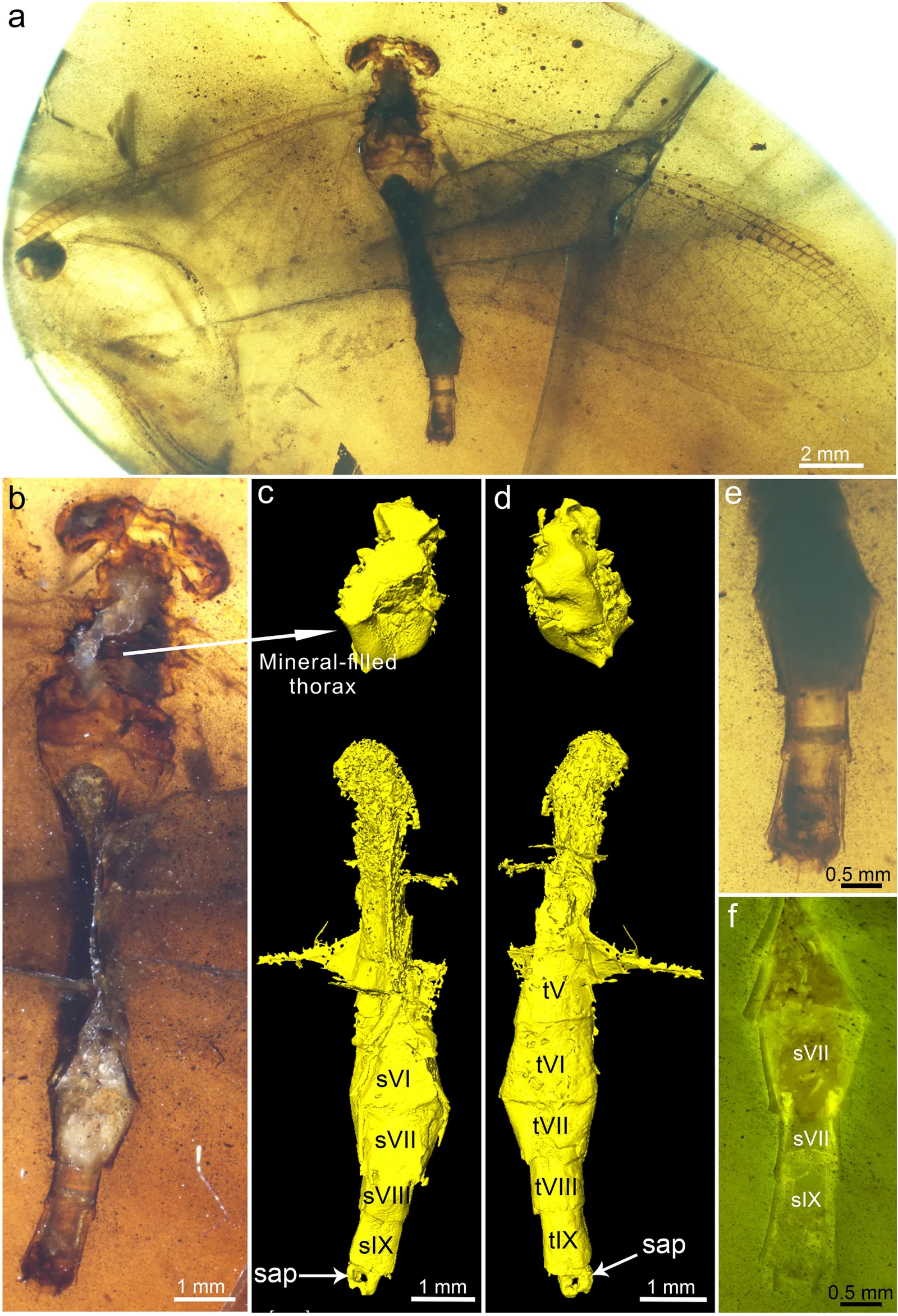
*Crehkahtihtengia dongi* gen. et sp. nov., female imago, holotype, NIGP207447. **a** Overview of body in dorsal view. **b **Reflected light micrograph of non-winged body in dorsal view. **c**, **d**. Microtomographic reconstruction showing the body filled with minerals, (**c**) in ventral view. (**d**) in dorsal view. e, Reflected light micrograph of posterior half of the abdomen.** f**. Fluorescent photograph of e, in ventral view.

**Diagnosis.** Body length 12.88 mm. Length-to-width ratio (L/W) of forewing ~ 3.1, hind wing length 0.45x forewing length. Adult of *Crehkahtihtengia* gen. nov. distinguished from other mayfly genera by the following combination of characters: *forewing* (a) relatively elongated, narrow, L/W ~ 3; basitornal margin relatively short, length 0.56x of tornoapical margin; (b) no gemination of longitudinal veins; (c) crossveins relatively numerous between C and RA; (d) MA forked proximally; (e) iCu field with four curved, loop-like triads, devoid of crossveins within triads; first two loop-like triads similar in width, second slightly wider than first, both wider than distal two; (f) CuP distally sharply bent, directed towards wing margin; (g) distal ends of CuA_2_ and CuP close; *hind wings* (h) MA forked proximally near mid-length of wing, slightly inclined towards margin; (i) iMA well developed.

Crehkahtihtengia dongi Stagg, Jiang, Staniczek & Godunko gen. et sp. nov. LSID urn: lsid: zoobank.org: pub: E14F638C-8A06-4D25-8577-C6CC0DF2B825.

**Fig. 2:**
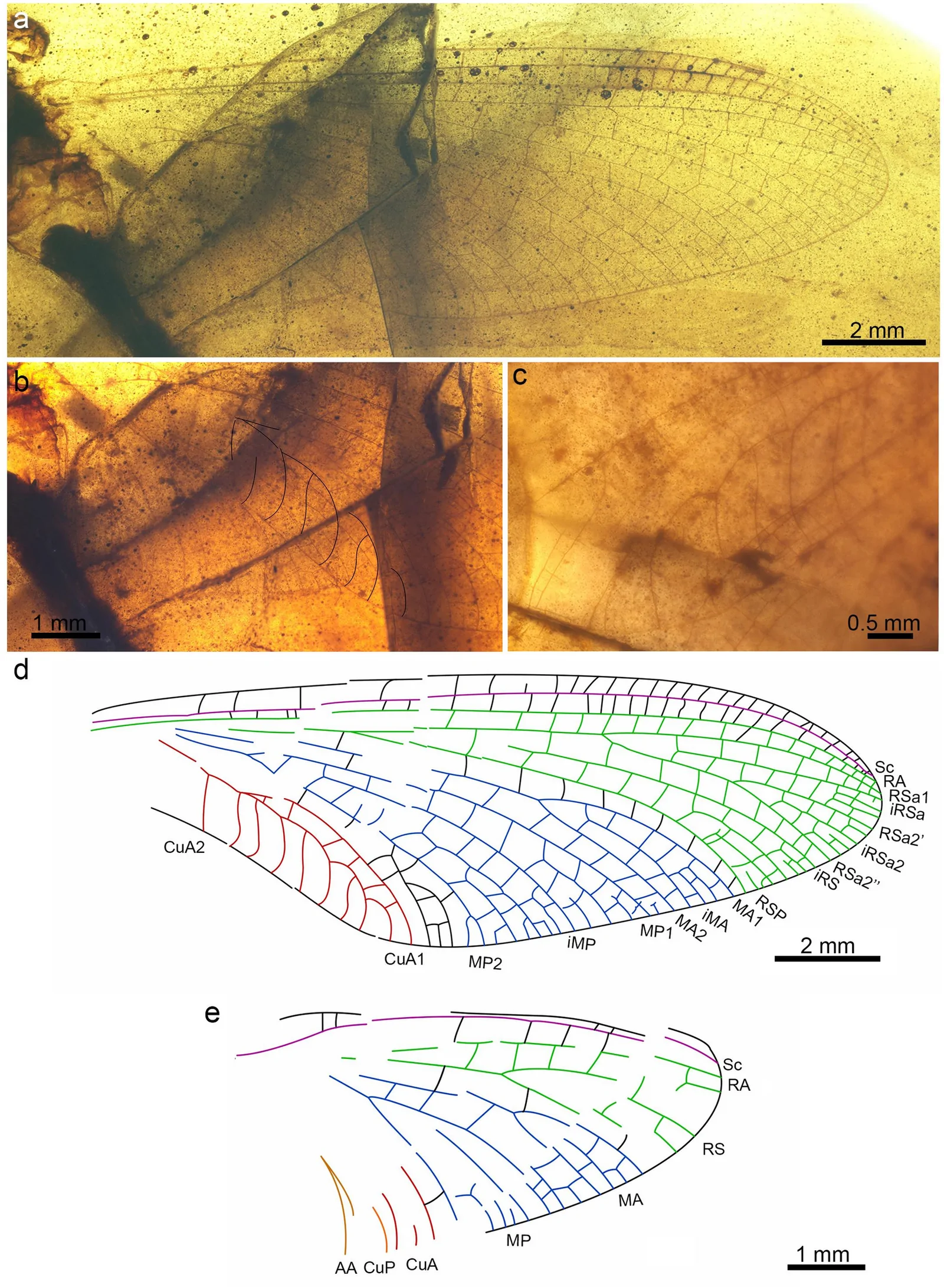
*Crehkahtihtengia dongi* gen. et sp. nov., female imago, holotype, NIGP207447. **a** Right forewing in ventral view. **b **Cubital field of right forewing in ventral view, four curved loop-like triads marked by black lines. **c **Cubital field of left forewing with four curved loop-like triads, ventral view. **d **Line drawing of right forewing in ventral view. **e **Line drawing of right hind wing in ventral view.

**Fig. 3:**
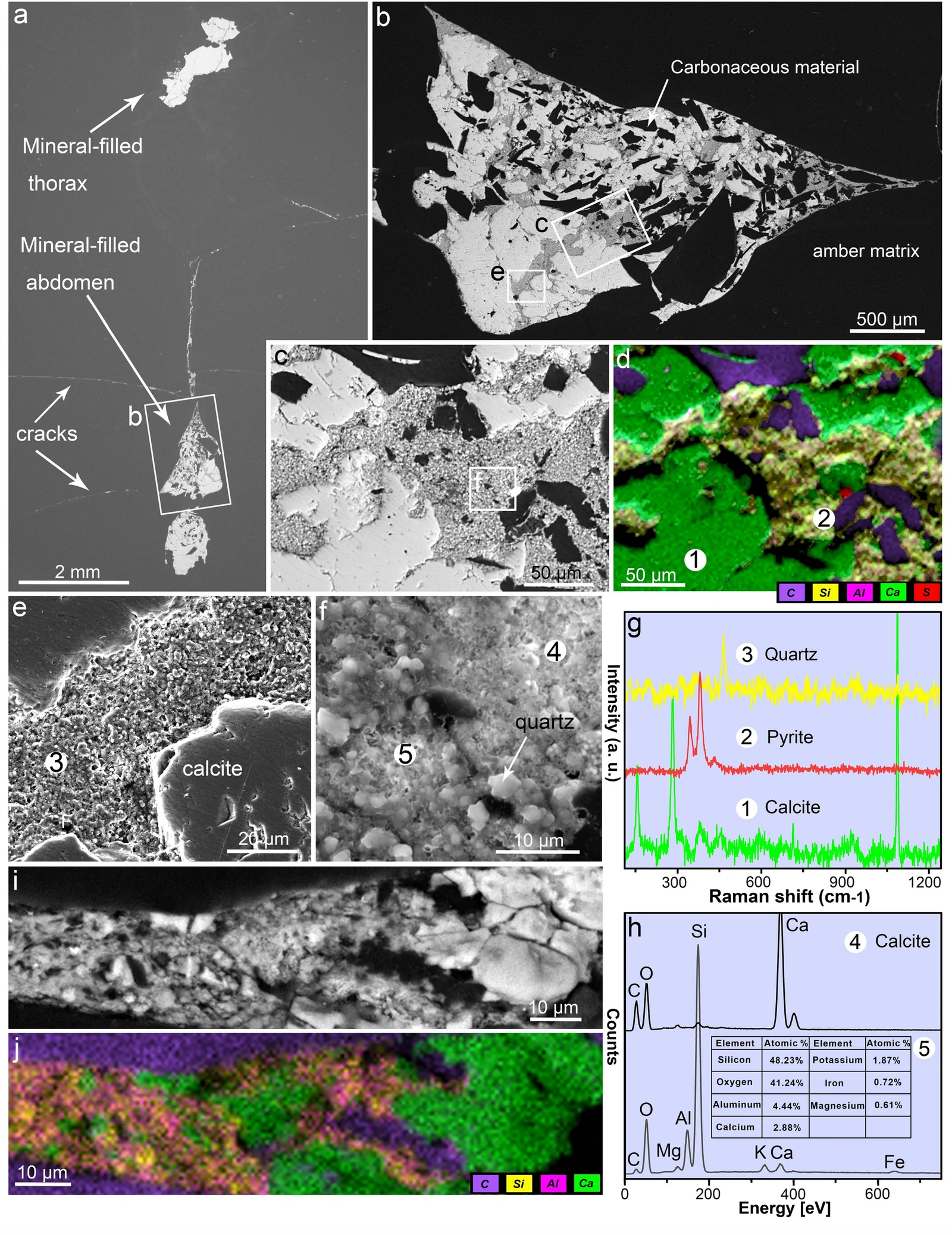
Taphonomic analysis of *Crehkahtihtengia dongi* gen. et sp. nov. NIGP207447 **a** Overview BSE image showing the exposed infilling mineral phases within the insect embedded in amber. **b** BSE image showing mineral phases exposed at abdominal segment VI. **c **BSE image of a selected region from panel b for detailed analysis. **d** EDS elemental map of c, with C (purple), Si (yellow), Al (pink), Ca (green), and S (red). **e** BSE image of a selected region from panel b, showing large euhedral calcite crystals and associated aggregates deposited within dissolution cracks in the calcite. **f **BSE image of the analysis area selected from c, showing fine-grained quartz crystals, microcrystalline calcite and iron-rich clay minerals. **g** Raman point spectra corresponding to the region shown in f. h, EDS point spectra from the region shown in e. **i**. BSE image showing mineral phases within cracks from a. **j **EDS elemental map corresponding to i, with C (purple), Si (yellow), Al (pink), Ca (green), and S (red).

**Fig. 4:**
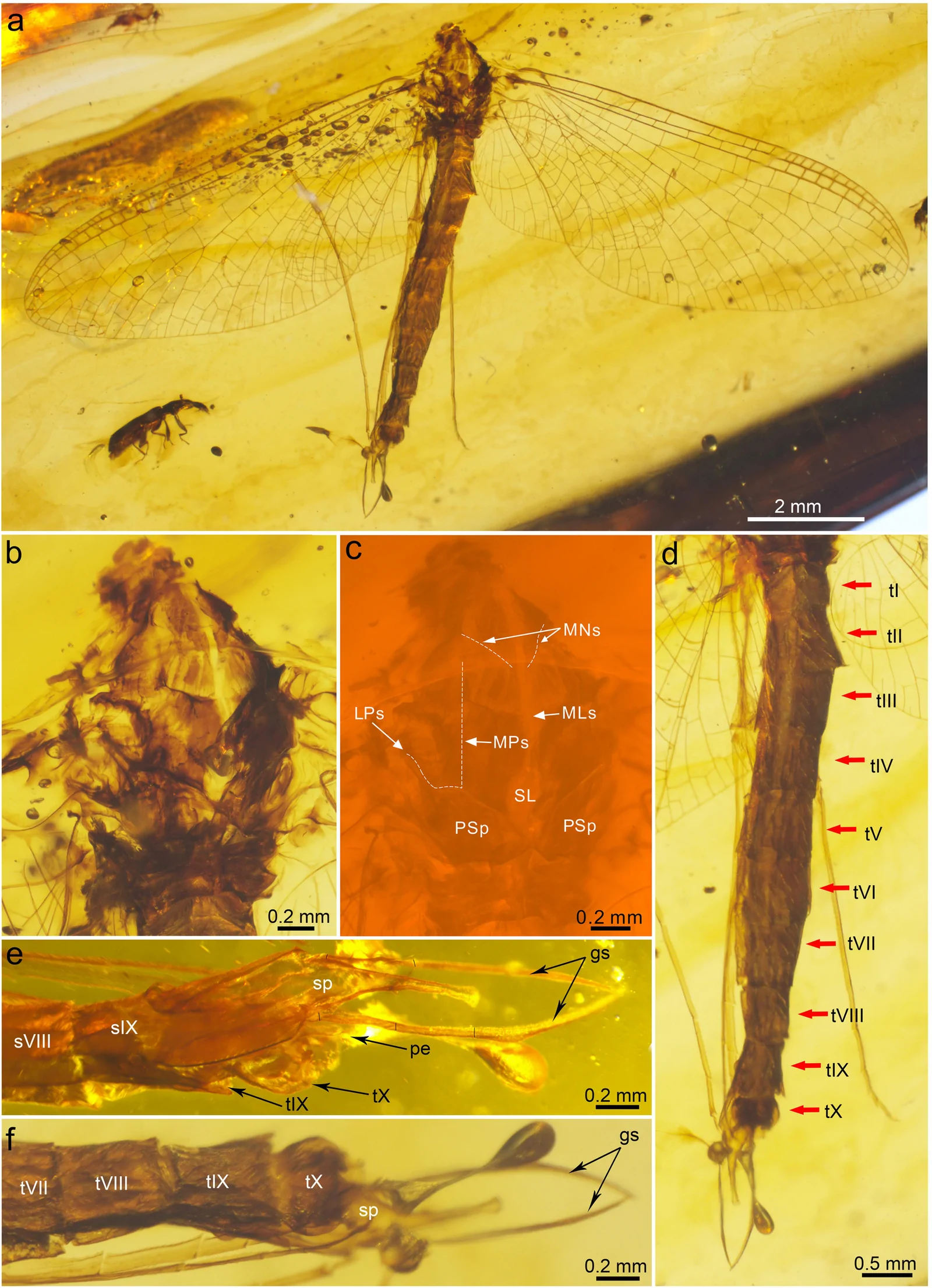
*Hexameropsis fehrmannorum* sp. nov., male imago, holotype, NIGP207448. Overview of body in dorsal view. **a **Photograph in dorsal view. **b** Thorax in dorsal view. **c **Head and thorax in dorsal view with thoracic sutures indicated by dotted lines.** d** Abdomen in dorsal view. **e **Tip of abdomen with genitalia in ventrolateral view. **f **Tip of abdomen with genitalia in dorsolateral view.

 Figures 1, 2, 3 and 4.

**Etymology.** The species epithet is dedicated to Professor Zhiming Dong, a pioneer of Chinese vertebrate palaeontology, in appreciation of his remarkable contributions to dinosaur research.

**Material.** Holotype, NIGP207447, female imago. Deposited in the Nanjing Institute of Geology and Palaeontology, Chinese Academy of Sciences; Nanjing, China.

**Locality and horizon.** Noije Bum Hill, Hukawng Valley, Kachin State, Myanmar; mid-Cretaceous, Cenomanian.

**Diagnosis.** *Female imago*. As for *Crehkahtihtengia* gen. nov.; genus currently monospecific.

**Description.** *Head* relatively large and wide, ~ 1.0 mm long and 3.0 mm wide in dorsal view; compound eyes large and prominent; three ocelli preserved.

*Thorax* ~ 4.3 mm long, 2.4 mm wide, poorly preserved; dorsal and ventral thoracic structures not discernible.

*Wings*. Forewing triangular, length ~ 16.8 mm, width ~ 5.3 mm, L/W ~ 3.3; basitornal margin relatively short, length 0.56x tornoapical margin; crossvenation well-developed; no gemination of longitudinal veins. RA: unbranched; basal two-thirds nearly straight, tapering towards apex. RS system: three branches, forming three triads; RSa_1_, RSp triad at 0.33 wing length from base; RSa_2_’’ contacting RSa_1,_ triad at 0.64 wing length from base; RSa_2_’ contacting RSa_2_’’, triad at 0.81 wing length from base; RSp unbranched. MA: forked into MA_1_ and MA_2_ at 0.56 wing length from base; iMA close to MA_2_; two short intercalaries between MA_1_ and iMA. MP: forked into MP_1_ and MP_2_ at 0.20 wing length from base; iMP slightly curved; several short intercalaries in MP field. CuA: bifurcated into CuA_1_ and CuA_2_ at 0.15 wing length from base, iCu: arising from CuA_1_ near base of fork, forming four curved, loop-like triads; successive triads connected, each contacting anterior one. Triads: lacking internal crossveins, first two triads nearly equal in width, second only slightly wider than first, both wider than distal two. Anal venation poorly discernible.

*Hind wings* elongated, preserved protion length ~ 7.6 mm, width ~ 3.35 mm. Sc and RA field broad. RS system with two branches; RA and RSp separation near MP fork; RS fork near wing midpoint. MA forked into MA_1_ and MA_2_ at distal quarter; iMA closer to MA_1_ than MA_2_. MP forked into MP_1_ and MP_2_ at basal quarter. Cubital and anal fields poorly preserved; veins poorly discernible.

*Legs* not preserved.

*Abdomen*. Segments I–VII mostly deformed, flattened, and fragmented in different planes; sternite VII damaged; traces of female external genitalia not preserved. Segment VIII shorter than IX. Terga VIII and IX with posterolateral projections. Subanal plate with a broad V-shaped incision.

**Preservation.** The specimen of *Crehkahtihtengia dongi* gen. et sp. nov. shows pervasive mineral infilling. Micro-CT images and backscattered electron (BSE) reveal that minerals extensively occupy the body cavity, particularly in the thoracic and abdominal regions (Figs. 1c and d and 3a). In BSE images, the fossil is markedly brighter than the surrounding amber matrix (Fig. 3a–c), indicating a strong compositional contrast. This contrast suggests that the body-cavity infill is dominated by minerals with a higher average atomic number (Z) than the amber matrix. In Fig. 3b and c, the dark outer region of the abdomen is bounded by the amber matrix, while the inner dark region consists of carbonaceous material, probably derived from organic residues of the fossil. Combined BSE images, EDS and Raman spectroscopy indicate that the predominant infilling minerals are calcite (CaCO_3_) and quartz (SiO_2_), with minor dispersed pyrite (FeS_2_), and clay-sized mineral aggregates (Fig. 3c–h). Calcite occurs as crystals up to ~ 500 μm in size, as well as fine-grained microcrystalline aggregates (Fig. 3c–h). Fine-grained quartz, typically < 5 μm, and is commonly associated with even finer phyllosilicate aggregates (Fig. 3c–f). These aggregates display a clay-sized texture and yield EDS signals of Si, Al, Fe, Mg, and K (Fig. 3e, f, h), but no diagnostic Raman signals were obtained. Semi-quantitative EDS analysis indicates a silica-rich phase (quartz or amorphous silica) with minor Al–K–Ca, possibly reflecting admixture of clay/aluminosilicate material and/or carbonates. (Fig. 3h). The presence of K in the EDS spectra further indicates that these clay-sized mineral aggregates may also include K-bearing phyllosilicates. Minerals are also observed filling fractures and cracks in the amber. BSE imaging and EDS elemental maps show that the crack fills are dominated by Si, Al, and Ca, consistent with the major infilling minerals within the fossil, including calcite, quartz, and aluminosilicates (Fig. 3i, j). This suggests a similar mineral composition in the crack fill and in the material infilling the fossil body cavity. Although the specimen is predominantly infilled, traces of organic matter are still preserved.

Genus ***Hexameropsis*** Tshernova & Sinitshenkova, 1974^41^, ^94^.

Type species: *Hexameropsis selini* Tshernova & Sinitshenkova, 1974^41^, ^94^.

***Hexameropsis fehrmannorum*** Stagg, Jiang, Staniczek & Godunko sp. nov.

Figures 5, 6 and 7.

*LSID* urn: lsid: zoobank.org: pub: E14F638C-8A06-4D25-8577-C6CC0DF2B825.

**Etymology.** The species epithet *fehrmannorum* is dedicated to first author’s maternal grandparents, Erika and Rudolf Fehrmann, in appreciation of their role in nurturing the first author’s love of the natural world since childhood.

**Material.** Holotype, NIGP207448, male imago. The holotype is deposited in the Nanjing Institute of Geology and Palaeontology, Chinese Academy of Sciences; Nanjing, China.

**Locality and horizon.** Noije Bum Hill, Hukawng Valley, Kachin State, Myanmar; mid-Cretaceous, Cenomanian.

**Diagnosis.** Moderately sized specimen, body length ~ 7.8 mm. L/W of forewing ~ 2.4; basitornal margin relatively long, 0.66x tornoapical margin length; hind wings 0.43x forewing length. The male imago of *H. fehrmannorum* sp. nov. differs from *H. elongatus* by the following characters: *hind wings*: MA fork located more proximally, iMA well developed (vs. *H. elongatus*, with MA fork more distally; iMA undeveloped); *genitalia*: unpaired median process of styliger elongate, slender, 0.57x of sternite IX length; gonostyli 5-segmented; penes robust, stick-shaped (vs. *H. elongatus* holotype, median process of styliger 0.46x of sternite IX length; gonostyli 4-segmented; penes slim, stick-shaped).

**Fig. 5:**
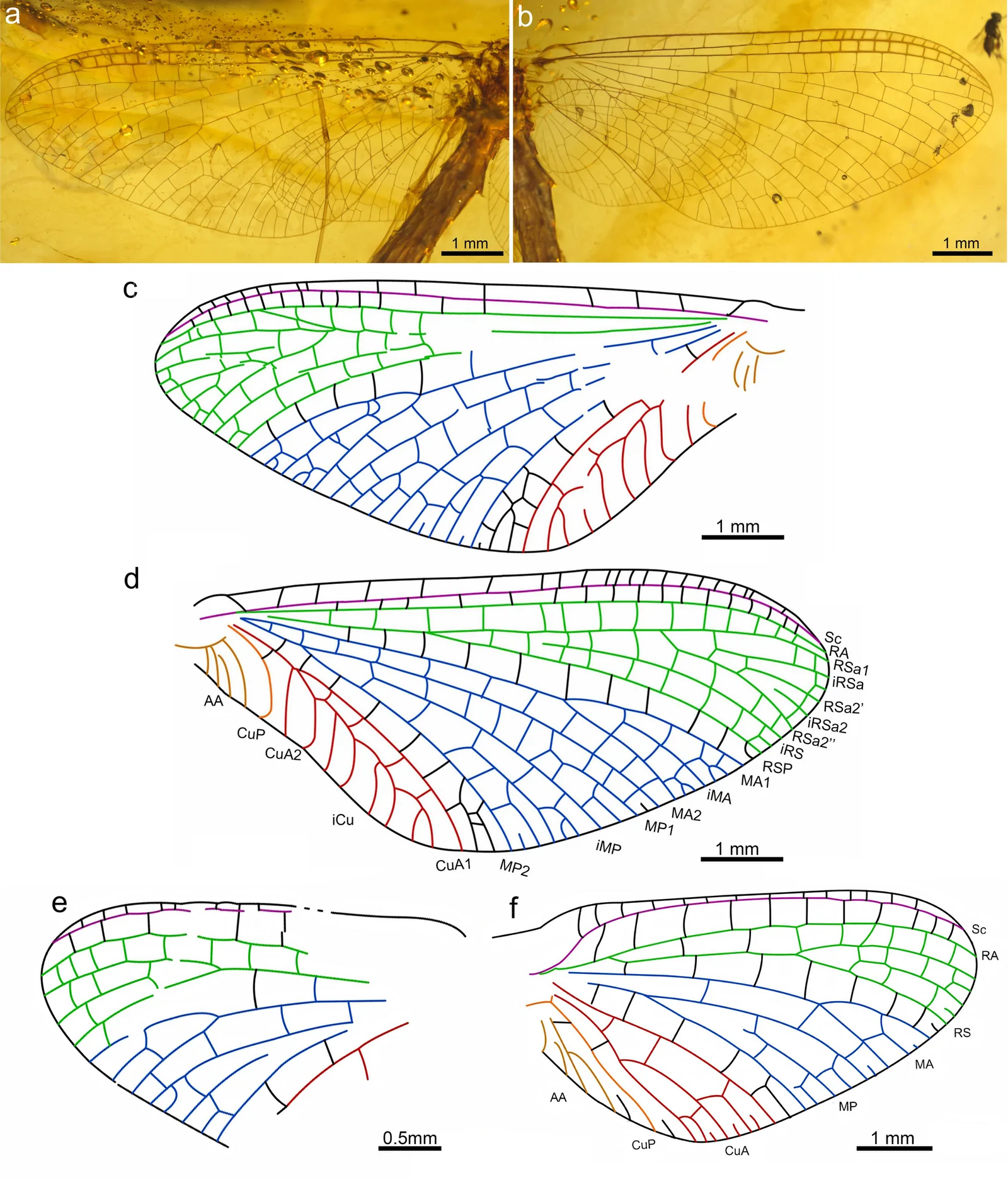
*Hexameropsis fehrmannorum* sp. nov., male imago, holotype, NIGP207448. **a **Photograph of left forewing. **b **Photograph of right forewing.** c** Line drawing of left forewing. **d** Line drawing of right forewing. **e **Line drawing of left hind wing. **f **Line drawing of right hind wing.

**Description.** *Head* ~ 0.3 mm long, ~ 0.46 mm wide; head and eyes partly damaged and incomplete.

**Fig. 6:**
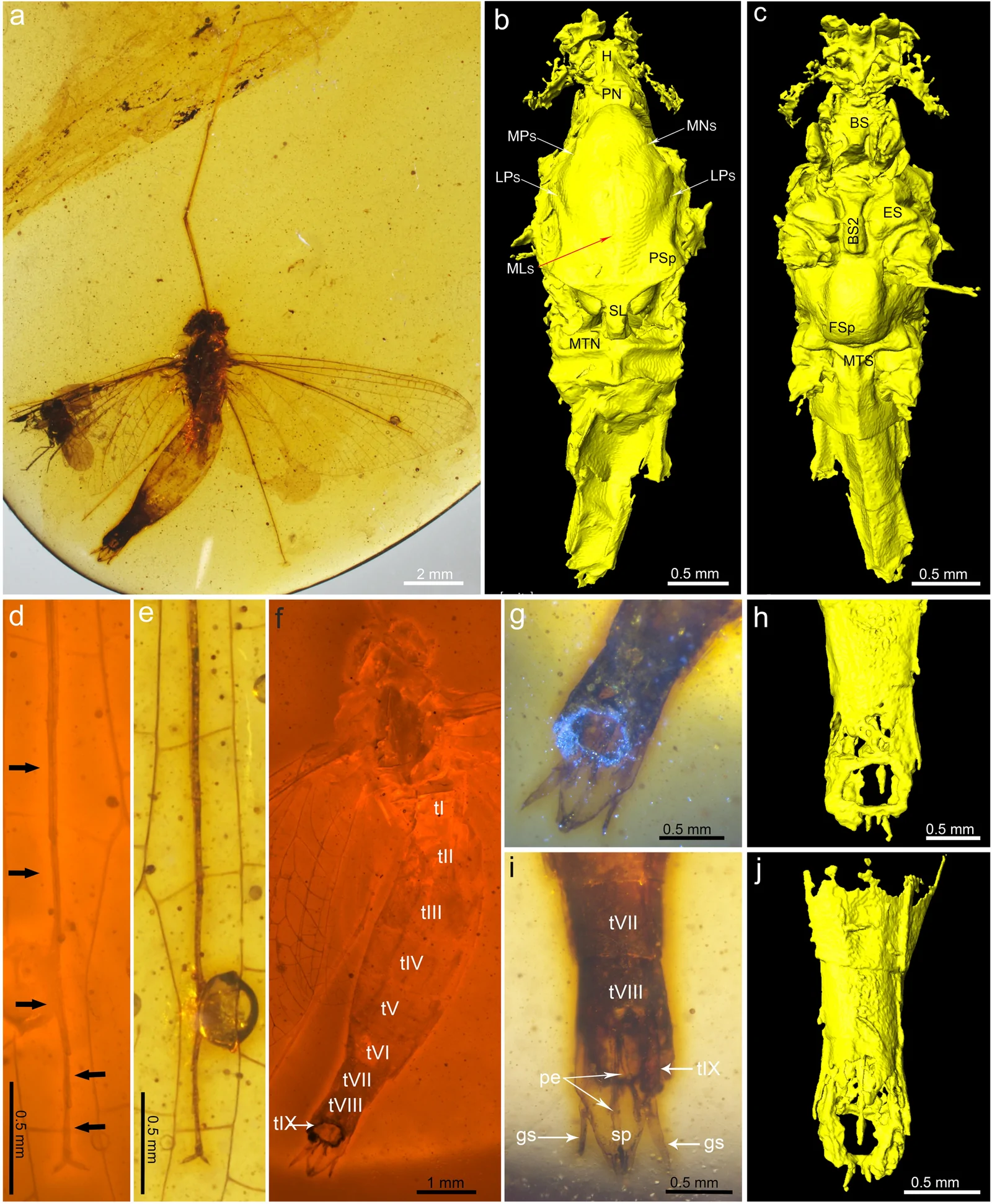
Hexameropsis cf. elongatus, male imago, NIGP207449. **a** Photograph, overview in dorsal view. **b** Microtomographic reconstruction, showing head, thorax and last three segments of abdomen in dorsal view. **c** Microtomographic reconstruction of head, thorax and first three abdominal segments in ventral view. **d** Tibia and tarsus of left middle leg under fluorescence microscope, ventral view. **e** Tibia and tarsus of left middle leg in dorsal view. **f** Fluorescent microphotograph of non-winged body in dorsal view, showing abdominal segments. **g** Tip of abdomen in dorsal view, reflected light micrograph. **h **Tip of abdomen in dorsal view, microtomographic reconstruction. **i** Tip of abdomen in ventral view, reflected light micrograph. **j **Tip of abdomen in ventral view, microtomographic reconstruction.

*Thorax* ~ 1.6 mm long, ~ 1.2 mm wide at widest. Prothorax narrow. Mesonotum as in Fig. 6D. Thoracic sterna not preserved.

*Forewing* triangular, length ~ 8.2 mm, width ~ 3.3 mm; L/W ratio ~ 2.4; costal brace distinct; crossveins well developed. Costal margin curved, tapering towards the apex; outer (tornoapical) margin longer than the anal (basitornal) margin; basitornal margin relatively long, 0.66x tornoapical margin length. RA fused with Sc in basal part of wing; RA subparallel to Sc, unbranched; basal two-thirds nearly straight, then tapering towards wing apex. RS system branched twice, forming two triads; RSa_1_ and RSp form a triad at about the anterior third of the wing length from the base; RSa_2_’’ contacts RSa_1_ in the distal quarter of the wing, near the wing margin, forming a triad; RSp simple. MA forked into MA_1_ and MA_2_ at 0.59 wing length from base; iMA approaching MA_1_ and MA_2_. MP basally forked into MP_1_ and MP_2_ at 0.19 of wing length; iMP slightly curved. CuA basally forked into CuA_1_ and CuA_2_ at 0.16 of wing length from base; iCu arising from CuA_1_ close to base of fork, forming three loop-like triads, first two triads subequal in width, each with an additional intercalary vein; third triad smallest; all branches reaching basitornal margin of wing.

*Hind wing* Length ~ 3.6 mm, width ~ 2.2 mm; venation moderately developed. Costal process distinct. Sc and RA field large. RS system with two branches, forming two triads: RSa_1_ and RSp forming first triad at 0.23 wing length from base, RSa_2_’’ contacting RSa_1_ to forming second triad at 0.63 wing length from base. MA distally bifurcated into MA_1_ and MA_2_ at 0.68 wing length from base; iMA close to MA_1_ and MA_2_. MP forked into MP_1_ and MP_2_ at 0.32 wing length from base. Cubital triad well developed, with small intercalaries between main longitudinal veins; CuP ending close to tornus. Five anal veins identifiable.

*Legs* long and slender. Left foreleg partially preserved, length ~ 5.0 mm; tarsomere I–V length ~ 1.0/1.2/1.0/0.6/0.3 mm; left midleg partially preserved, length ~ 5.3 mm; left hind leg partially preserved, length ~ 5.7 mm; right hind leg partially preserved, length ~ 3.7 mm in dorsal view. Right foreleg: tarsus strongly elongate, five segments; tarsomeres I–V length ~ 0.48/0.43/0.25/0.13/0.19 mm. Claws long, acute and hooked.

*Abdomen* length ~ 5.9 mm, width ~ 0.8 mm; elongate, cylindrical. L/W approximately consistent segments I–VIII. Ribbed texture resembling sphenopsid longitudinal ribs or striae, with horizontal nodes resembling segment margins. Abdominal sternite IX prolonged, ~ 2 length of tergum X.

*Genitalia* completely preserved; unpaired median process of styliger is contiguous with gonostyli, relatively elongate and slender, 0.57x length of sternite IX (measured from process tip to gonostyli base). Gonostyli preserved, both ~ 1.4 mm long, segmentation poorly defined, gonostylus 5-segmented; basal segment shortest; length ratios of gonostylus segments I–V: 0.15/1.00/0.70/1.50/0.25; penes stick-shaped, robust.

**Preservation.** As the fossil is not exposed from the surrounding amber matrix, compositional analysis was not performed.

***Hexameropsis cf. elongatus*** Lin et al., 2018.

Figures 8, 9.

**Material.** NIGP207449, imago, male. The specimen is deposited in the Nanjing Institute of Geology and Palaeontology, Chinese Academy of Sciences; Nanjing, China.

**Locality and horizon.** Noije Bum Hill, Hukawng Valley, Kachin State, Myanmar; mid-Cretaceous, Cenomanian.

**Revised diagnosis **(after ref^23^.). Moderately sized specimen, body length ~ 10.2 mm. Forewing L/W ~ 2.2, hind wings 0.42x forewing length. *Forewings*: basitornal margin relatively long, 0.76x tornoapical margin length; iCu field with 2–3 curved loop-like triads of varying widths; first triad widest; crossveins within triads absent or weak; *Hind wings*: MA forked at quarter length near margin; iMA absent. *Genitalia*: penes slim, stick-shaped.

**Fig. 7:**
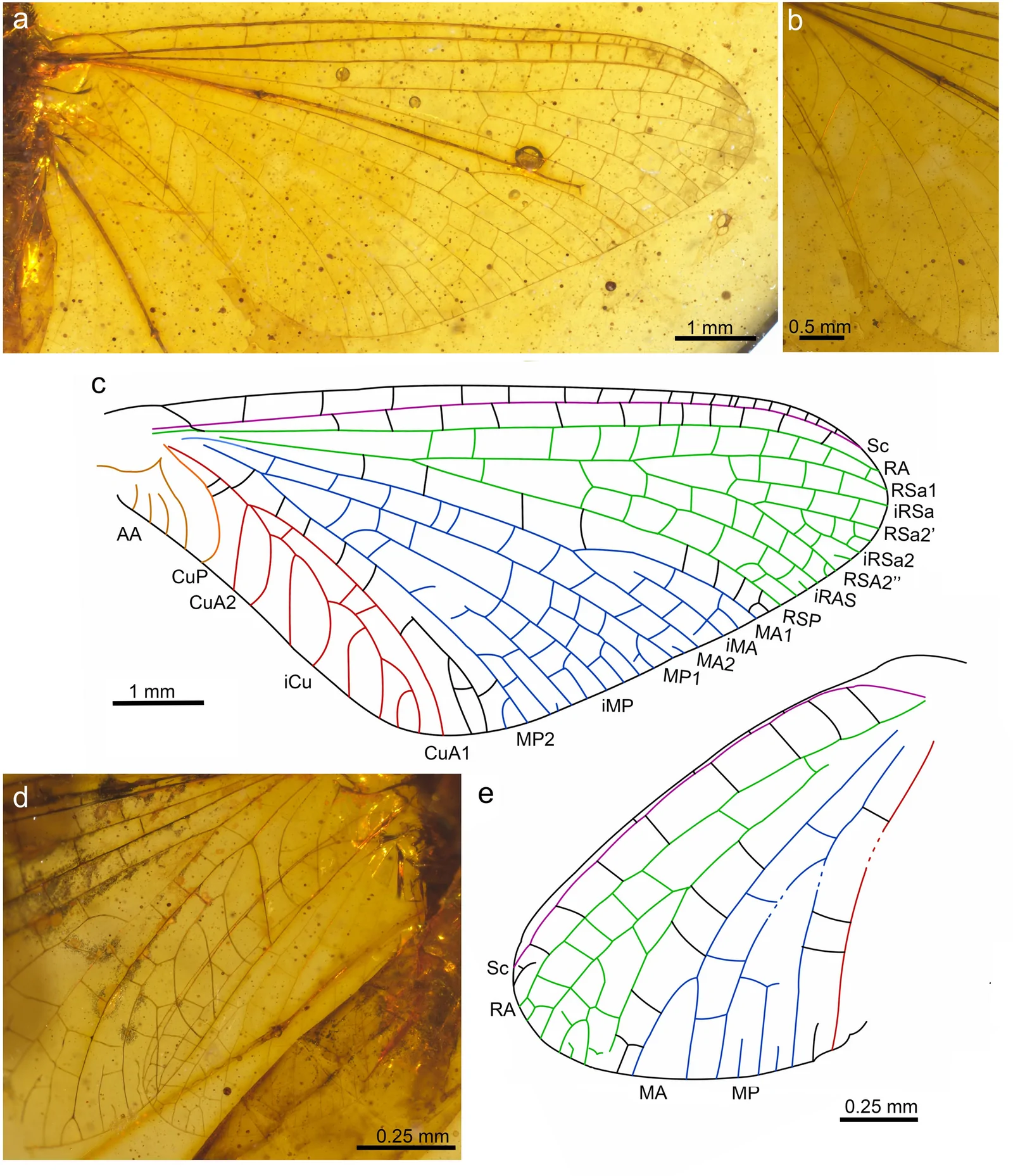
Hexameropsis cf. elongatus, male imago, NIGP207449. **a** Photograph of right forewing. **b** Cubital field of right forewing showing three curved, loop-like triads. **c** Line drawing of right forewing. **d** Line drawing of right hind wing. **e** Cubital field of left forewing showing three curved, loop-like triads, photograph.

**Description.** *Head.* ~0.7 mm long, ~ 1.5 mm wide; large compound eyes preserved.

*Thorax* ~ 2.6 mm long, ~ 1.5 mm wide. Prothorax broad. Mesonotum as in Fig. 9B; mesosternum as in Fig. 9C.

*Forewing* triangular, length ~ 8.8 mm, width ~ 3.9 mm, L/W ~ 2.3; costal brace distinct; crossveins well developed. Costal margin curved, tapering towards apex; outer (tornoapical) margin longer than anal (basitornal) margin; posterior margin slightly concave; costal field broad at base, narrowing towards tip. RA subparallel to Sc, forming a broad field; width more than half between RA and RSa_1_; basal two-thirds nearly straight, apically tapering; RA unbranched. RS system with three branches, forming three triads; RSa_1_, RSp triad at 0.40 wing length from base; RSa_2_’’ contacts RSa_1_ triad at 0.68 wing length from base; RSa_2_’ contacting RSa_2_’’, triad at 0.83 wing length from base; RSp unbranched. MA branched into MA_1_ and MA_2_ at 0.59 wing length from base; iMA approaching MA_1_ and MA_2_. MP branched into MP_1_ and MP_2_ at 0.21 wing length from base; iMP slightly curved. CuA forked into CuA_1_ and CuA_2_ at 0.18 wing length from base; iCu arising from CuA_1_ close to base of fork; three loop-like triads in left forewing, two in right; first triad widest, crossveins within triads absent or weak.

*Hind wing.* Preserved part ~ 3.7 mm long, ~ 2.5 mm wide. Costal process distinct. Sc and RA field broad. RS system with two branches. MA bifurcated into MA_1_ and MA_2_ near distal quarter. iMA approaching MA_1_ and MA_2_. MP bifurcated into MP_1_ and MP_2_ near wing midpoint.

*Legs* slender, elongate. Right foreleg length ~ 13.8 mm; right midleg length ~ 6.8 mm; right hind leg slightly shorter, length ~ 6.3 mm; left hind leg preserved length ~ 5.2 mm. Foreleg femur and tibia subequal in length. Tibia spurs present on mid- and hind legs. Foreleg tarsi elongate, comprising ~ 1/3–1/2 of total leg length; tarsomeres II–IV unusually long. Midleg tarsomeres subequal in length. Claws of right mid- and hind legs hooked, slightly curved.

*Abdomen.* Length ~ 6.9 mm. Segments I–VIII and part of tergite IX preserved; tergite X absent. Segments III–VII deformed and dorsoventrally flattened. Cerci and traces of paracercus absent.

*Genitalia* damaged; distal part of unpaired median process of styliger and distal segments of gonostyli absent; penes slim stick-shaped; lobes well divided.

**Fig. 8:**
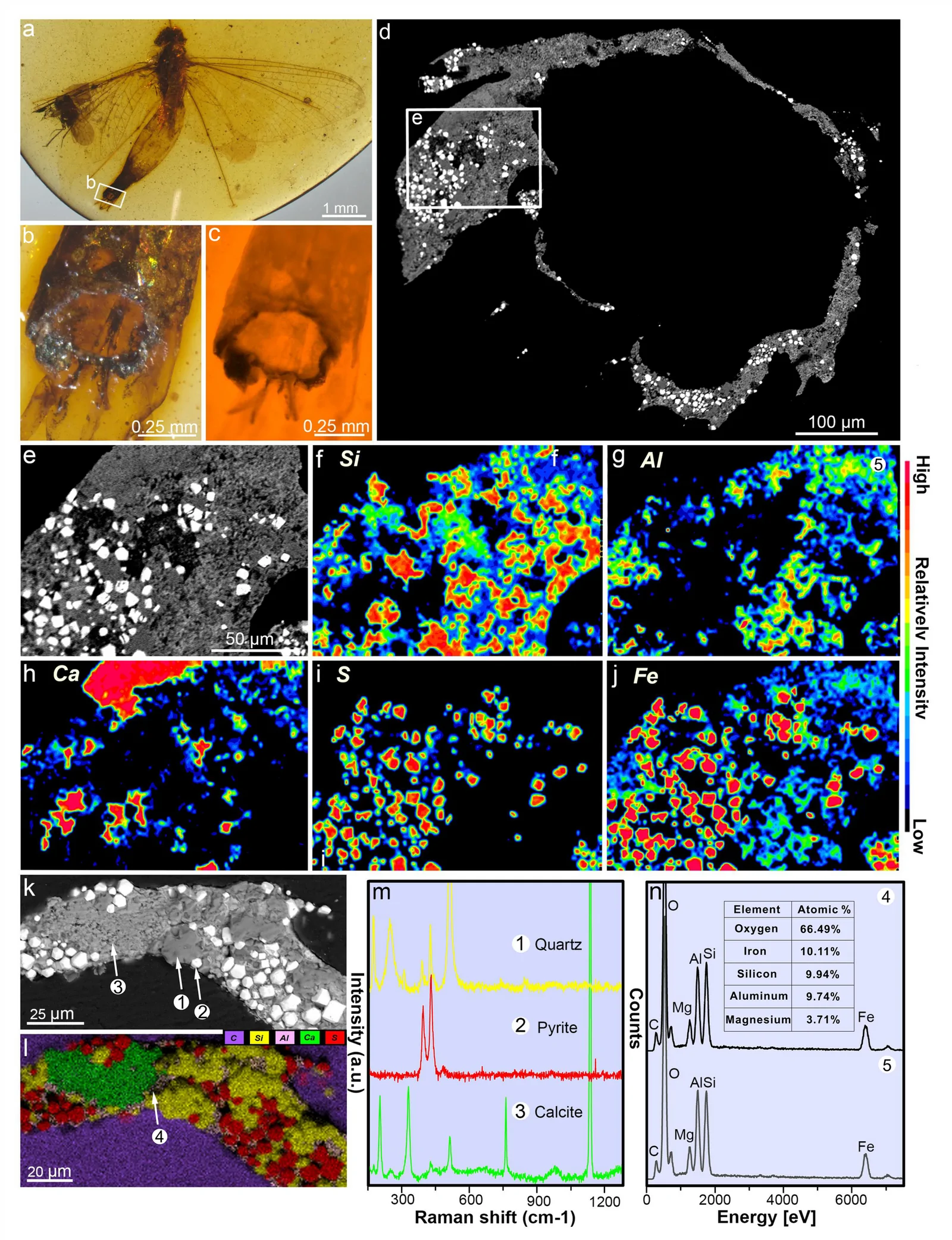
Taphonomic analysis of *H. cf. elongatus.* NIGP207449. **a** Overview showing the position of the analysed region at the terminal end of abdominal segment IX. **b** Reflective light micrograph of the localized analysis area. **c **Fluorescence light micrograph of the same region of b. **d **BSE image showing the overall mineralized region selected for analysis. **e** BSE image of a selected subregion from b for detailed analysis. **f–j**, WDX elemental maps showing the distribution of Si, Al, Ca, S, and Fe, respectively. **k**, BSE image of the area shown in b. **l**, EDS elemental map, with C (purple), Si (yellow), Al (pink), Ca (green), and S (red). **m**, Raman point spectra corresponding to the region in l. **n**, EDS point spectra from the region shown in e.

**Preservation.** *Hexameropsis cf. elongatus* exhibits mineral-filled preservation (Fig. 8). The 9th abdominal segment is exposed at the amber surface (Fig. 8a–d), allowing both morphological and compositional analyses of the exposed area using SEM, EDS and wavelength-dispersive X-ray spectroscopy (WDX) (Fig. 8b–n). In the BSE images, the brightest particles correspond to pyrite (Fig. 8b, e, k), confirmed by EDS/WDX and Raman, and occurr as euhedral to subhedral crystals with well-defined boundaries and grain diameters generally ranging from ~ 2 to 13 μm. These pyrite crystals are prominently highlighted in the WDX elemental map by intense red coloration for sulfur (S) and iron (Fe), and in the EDS elemental map by intense red coloration for sulfur (S) (Fig. 8i, j, l). Raman spectroscopy further confirms that these particles are pyrite (Fig. 8m). Calcite occurs as dense microcrystalline aggregates corresponding to Ca-enriched zones in the WDX and EDS maps (Fig. 8h, l); its presence is further confirmed by Raman spectroscopy (Fig. 8m). Quartz occurs locally as microcrystals, typically ranging from 0.5 to 2.5 μm in size, and as compact, blocky crystal with well-defined faces up to ~ 20 μm in diameter (Fig. 8e, k). Quartz-rich areas in the WDX and EDS elemental maps correspond to regions enriched in silicon (Si) and relatively depleted in aluminium (Al) (Fig. 8f, l), with Raman spectroscopy further confirming the presence of quartz (Fig. 8m). SEM, WDX, and EDS results further indicate that some mineral aggregates show fine sheet-like textures or clay-sized dimensions, with elemental compositions containing Si, Al, Fe and Mg (Fig. 8e–g, n). No diagnostic Raman peaks were obtained from these aggregates. These aggregates appear polymineralic. Semi-quantitative EDS compositions tentatively suggest that the Fe-rich hydrous silicates are most consistent with chlorite-group minerals, most likely chamosite or the closely related berthierine, which is a serpentine-group mineral (Fig. 8n).

## Discussion

In this study, we report a new genus and species, *Crehkahtihtengia dongi* gen. et sp. nov., a new species *H. fehrmannorum* sp. nov., and a male imago of uncertain taxonomic status tentatively assigned to the genus *Hexameropsis*, *H. cf. elongatus*. All of these specimens originate from mid-Cretaceous amber of northern Myanmar and are assigned to the extinct Mesozoic mayfly family Hexagenitidae. Previously, only *Hexameropsis elongatus* had been reported from Burmese amber^23^. These new specimens are attributed to Hexagenitidae based on the distinctive forewing venation of the imago, including a branched CuA and multiple successive loop-like triads between CuA_1_ and CuA_2_. Among them, *Crehkahtihtengia* gen. nov. shows four successive loop-like triads between CuA_1_ and CuA_2_ in the forewings, whereas *Hexameropsis* features only two or three. Additionally, these extinct taxa embedded in fossil resins preserve additional characters, further improving our understanding of the taxonomic position of the new specimens and the morphological diversity within Hexagenitidae, namely thoracic structures.

*Crehkahtihtengia* gen. nov. is proposed in this study as a new genus, distinguished from other genera of Hexagenitidae by the following features. *Crehkahtihtengia* gen. nov. is of moderate body size, with a forewing length of ~ 16.8 mm, ranging from 6.9 to 23 mm (key identification characters from Lin et al., 2018^23^. It differs from the large-sized Hexagenitidae preserved in limestone as compression fossils, including *Epicharmeropsis* and *Ephemeropsis*, which have forewing lengths ranging from 35 to 43 mm. *Epicharmeropsis* and *Ephemeropsis* possess four to six curved, loop-like triads in the cubital field, but exhibit more crossveins and intercalary veins between and within the triads than other genera of Hexagenitidae, whereas *Crehkahtihtengia* gen. nov. lacks crossveins within the triads in the cubital field. Additionally, the longitudinal veins of *Crehkahtihtengia* gen. nov. are not geminate, in contrast to *Epicharmeropsis* and *Ephemeropsis.* The presence of four curved, loop-like cubital triads distinguishes *Crehkahtihtengia* gen. nov. from *Mongologenites* Sinitshenkova, 1986, which has six loops, and from *Hexameropsis*, which has three. *Cratogenitoides* and *Hexagenites* likewise have four looped triads in the cubital field; however, *Cratogenitoides* can be distinguished by the presence of numerous cubital crossveins and a significantly shorter hind wing. In contrast, *Hexagenites* exhibits less developed crossveins in the cubital field of the forewing and a hind wing nearly as long as the forewing, distinguishing it from both *Cratogenitoides* and *Crehkahtihtengia* gen. nov.

*Hexameropsis* was previously represented in mid-Cretaceous Burmese amber by two species, *H. selini* and *H. elongatus*. *Hexameropsis fehrmannorum* sp. nov. and *H. cf. elongatus*, both have significantly fewer crossveins and intercalary veins in the forewing compared to *H. selini*. The forewing lengths of *H. fehrmannorum* sp. nov. and *H. cf. elongatus* are similar to each other but much shorter than the ~ 20 mm forewing length of *H. selini*. In *H. fehrmannorum* sp. nov. and *H. cf. elongatus*, the third curved loop-like triad in cubital field of forewing is smaller than the other two, whereas it is wider in *H. selini*. *H. fehrmannorum* sp. nov. differs from *H. cf. elongatus* in having the MA fork of the hind wing positioned more proximally and a well-developed iMA. *H. cf. elongatus* exhibits a similar body and wing size, as well as wing venation, and the hind wings lacks iMA, consistent with *H. elongatus*.

Besides wing venation as an important identifying feature, genitalia are also a crucial for identification. However, in the holotype of *C. dongi* sp. nov. (female imago), sternite VII is damaged, and traces of female external genitalia are not preserved. Nevertheless, the characteristic shape of the subanal plate in *C. dongi* sp. nov., which is broadly incised apically, may serve as a diagnostic feature of *Crehkahtihtengia* gen. nov. in the future, when additional females of Hexagenitidae are discovered in Burmese amber.

New fossils further reveal the variability and distinctiveness of genital structures in Burmese amber, contributing to future studies on the evolutionary development of these features as more fossil data accumulates. A characteristic feature of *Hexameropsis* is its elongated abdominal sternum IX, which tapers towards the apex and is variable in size and shape among known males of this genus, distinguishing it from other hexagenitid fossils that preserve abdominal sternum IX^23,33^. The male genitalia of *H. fehrmannorum* sp. nov. and *H. elongatus* differ markedly in the structure of styliger plate, including the length ratio of unpaired median process of styliger to sternite IX, and in the number of distal segments of gonostyli. The two species can also be separated by the shape of penis lobes. Detailed comparison of genitalia of *H. elongatus* and *H. cf. elongatus* is difficult because the genitalia are damaged in the latter. Nevertheless, in addition to similarities in size and wing venation, both taxa have penis lobes that are stick-like, relatively slender, well separated, and laterally divergent (see above). Thus, based on the similarity in wing venation and the shape of penes, we tentatively assign the male imago with inventory number NIGP207449 to *H. cf. elongatus*.

*Crehkahtihtengia* gen. nov. is the second genus of Hexagenitidae described from Burmese amber, and it is currently known from the single species, *C. dongi* sp. nov. In addition to differences in the number of loop-like triads in the cubital field of the forewing, *Crehkahtihtengia* gen. nov. also differs from *Hexameropsis* in other features, including a significantly larger body and forewing size; the forewing of the female imago of *C. dongi* sp. nov. is relatively narrow, and nearly twice as long as that of species of *Hexameropsis*. In terms of forewing shape, *C. dongi* sp. nov. has a well-developed, elongate tornoapical margin, whereas *H. elongatus*, *H. cf. elongatus*, and *H. fehrmannorum* sp. nov. exhibit a more triangular forewing, with the tornus located farther from the costal margin (the anterior wing margin). In addition, the forewing MA forks more proximally in *C. dongi* sp. nov., whereas it forks more distally in *H. elongatus*, *H. cf. elongatus*, and *H. fehrmannorum* sp. nov.; forewing CuP is distally straight in *C. dongi* sp. nov., but in *Hexameropsis* the distal end of CuP is sharply bent at its contact with the wing margin; the distance between the distal ends of CuA_2_ and CuP in the forewing of *C. dongi* sp. nov. is smaller than that in species of *Hexameropsis*.

**Fig. 9:**
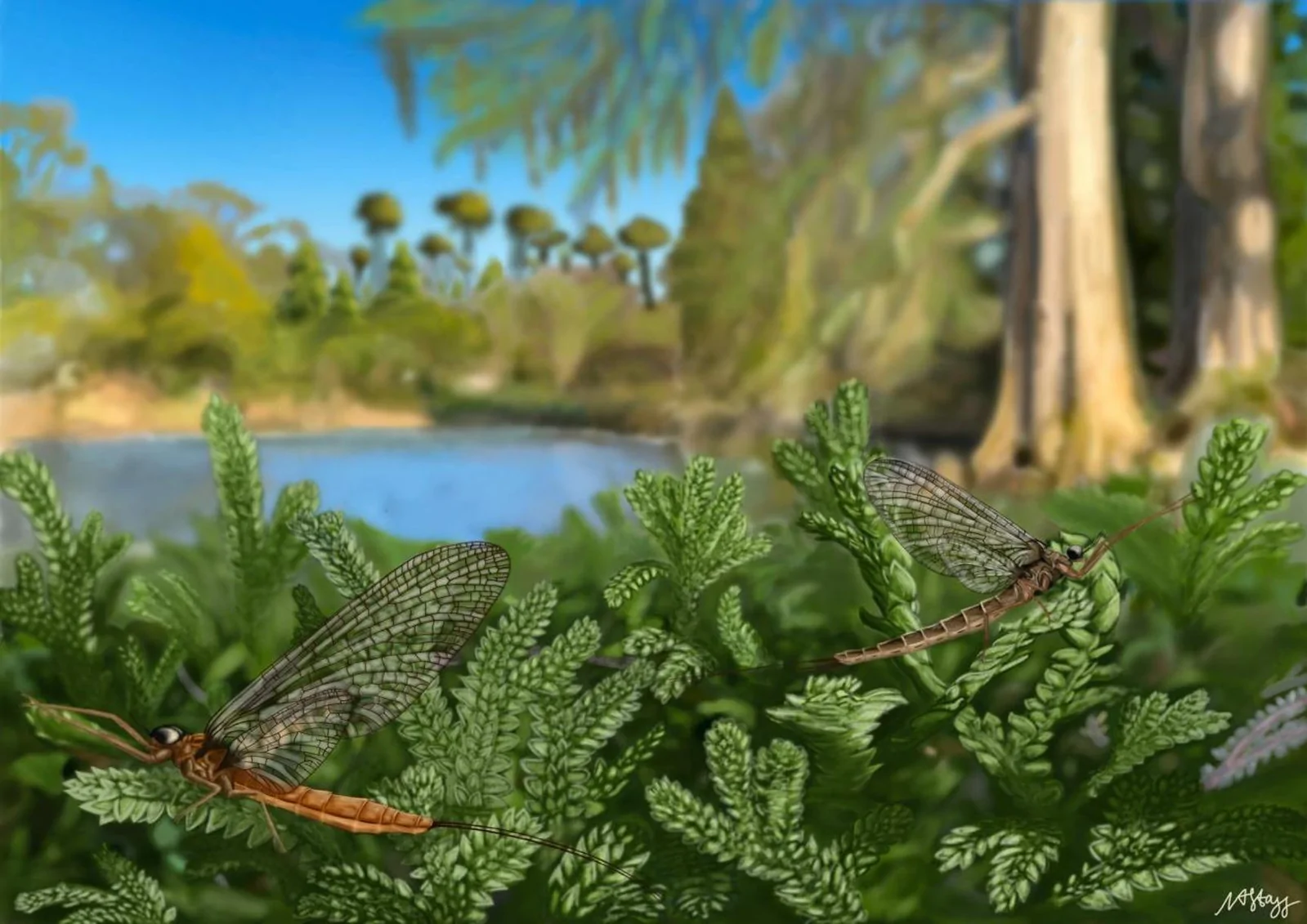
Life and ecological reconstruction of *Crehkahtihtengia dongi* gen. et sp. nov. (left), and *Hexameropsis fehrmannorum* sp. nov. (right) in a mid-Cretaceous Kachin amber forest, illustrated by Nicolas Adrian Stagg.

### Palaeoecological implications

Reconstructing ecological patterns of adult Hexagenitidae in mid-Cretaceous Burmese amber is difficult. This family belongs to an entirely extinct Mesozoic lineage, and some included genera are known only from a single developmental stage. Therefore, unlike other ephemeropteran families recorded from Burmese amber (e.g., Vietnamellidae, Heptageniidae and Leptophlebiidae, Isonychiidae), which occur in the modern Oriental fauna and are known from both adults and larvae with relatively well-studied ecological preferences, Hexagenitidae from this deposit allow only a broad outline of palaeoecological conditions to be inferred.

On the other hand, given their early origin, mayfly lineages are generally conservative in larval distribution, with very low vagility comparison to other taxa. Individual species have different ecological requirements, which are reflected in their distributions and habitat preferences. For example, while only a few extant mayfly species inhabit a wide range of habitats, most of them prefer a narrow range of ecological conditions^69^(Bauernfeind & Soldán, 2012). This restriction is largely due to the limited flight ability of Ephemeroptera, with the main paths of dispersal along streams and rivers being downstream drift of larvae and/or upstream compensatory flight of fertilized females. Consequently, these insects accumulate in isolated benthic habitats only slowly, and colonization of new habitat types is usually a long-term process^70^. Because of the described fundamental ecological features of Ephemeroptera and in particular the Hexagenitidae, both larvae and adults can be used to analyse specific formations (horizons) or fossil resin. They are an important source for palaeoecological reconstructions, because, in most cases, they represent autochthonous fauna^71–73^.

Described Hexagenitidae have mostly been discovered in paleolake deposits, suggesting that these mayflies were primarily adapted to the lacustrine or shallow lacustrine-wetland paleoenvironment^33,74–78^. However, there is currently no evidence for the presence of comparable ecosystems within the proposed Burma Terrane during the Cretaceous. Rather, the opposite is true: the rich mayfly fauna documented in Burmese amber belongs to the families that todoay are represented almost exclusively by taxa with rheophilic larvae, characteristic inhabitants of running-waters habitats of various types, ranging from mountain streams and rivers to lowland, even deltaic, reaches of large rivers. Therefore, as recently suggested by Godunko et al. (2025)^9^, Hexagenitidae and another extinct family, Australiphemeridae McCafferty, 1991, probably also had rheophilous larvae. These larvae may have inhabited the lower (deltaic) reaches of the waterflows on the Burma Terrane, corresponding to the modern hyporhithral to epipotamal zones of current waters. Comparable, presumably deltaic mayfly taxocenes have been described from the Jurassic Solnhofen Limestone of Germany^79^ and, in part, from the Lower Cretaceous Crato Formation in Brazil^78^.

### Taphonomic preservation

To date, mineralization associated with amber inclusions can preserve structures originally formed by soft tissues mainly through mineral replication or mineral infilling, and these mineral accumulations in amber, which preserve specific anatomical structures, are typically dominated by a single mineral phase, most often calcite, silica, or pyrite. This study provides evidence for the first time that multiple mineral phases co-occurring within the body cavity infill of a single inclusion of *C. dongi* sp. nov. and *H. cf. elongatus*, including calcite, silica, pyrite, and clay-sized phyllosilicate aggregates. Our results demonstrate that clay minerals, hydrous aluminium phyllosilicates, were involved in the preservation of biological structures in amber inclusions. The associated mineral assemblages suggest that structural preservation may record the superposition of multiple depositional events within a single inclusion, and our findings substantially expand the known diversity and complexity of void fills in amber.

Insects generally lack biomineralized tissues composed of the minerals noted above. Therefore, the mineralization and/or infilling of mayflies in Kachin amber likely depended on the availability of chemical species in the surrounding microenvironment. These chemical inputs most likely derived from the decay of soft tissues driven by microbial respiration and from external sourced fluids that transported chemical species from the surrounding environment^53,68,80–83^. In *C. dongi* sp. nov., minerals within the fracture connected to the fossil match those infilling the fossilized body cavity, supporting the interpretation of Jiang et al. (2022)^68^ that mineralizing chemical species likely entered the resin/amber through fractures, reached the inclusion and initiated mineralization, thereby indicating that Kachin amber does not always behave as a closed system throughout subsequent burial and post-entombment alteration.

Calcite and quartz are both present within the body cavities of *C. dongi* sp. nov. and *H. cf. elongatus* (Figs. 3 and 8). The precipitation of two minerals is strongly governed by ambient pH, yet they exhibit inverse solubility trends under alkaline conditions^84^. Microbial activity, particularly anaerobic respiration, can induce localized acidification, potentially shifting the mineralization pathway from calcite precipitation to silica deposition^53,85^. In the studied specimens, the onset of microbial sulfate reduction may have lowered the pH in the surrounding microenvironment, thereby promoting the dissolution of early-formed calcite and the subsequent precipitation of silica. The occurrence of both framboidal aggregates and minute isolated euhedral crystals of pyrite within these fossils provides supporting evidence for the past presence of microbial sulfate reduction (Fig. 8). This process generates hydrogen sulfide (H_2_S), which reacts with dissolved ferrous iron (Fe^2+^) to precipitate pyrite^82,83^. In addition to sulfate reduction, other anaerobic microbial metabolisms, such as dissimilatory iron reduction and methanogenesis, may have contributed to local pH fluctuations, thereby facilitating both the remobilization of carbonate and the precipitation of silica phases.

In the studied mayfly fossils, we report the coexistence of abundant aggregated minerals rich in Si, Al, Fe, Mg, and K found together with other minerals–including calcite, quartz, and pyrite–within the fossil body cavities. These aggregated minerals also exhibit either clay-sized texture or a layered structure and yield no detectable Raman signals. Based on the semi-quantitative EDS results, the Fe-rich hydrous silicates in *H. cf. elongatus* are most consistent with chlorite-group minerals, likely chamosite, or with the closely related berthierine, a serpentine-group mineral. The presence of K in the EDS spectra from *C. dongi* gen. et sp. nov further suggests that these mineral aggregates may also contain other associated clay minerals. Previously, Jiang et al. (2022)^68^ identified spherical aggregates with a layered structure, generally ranging from 2 to 15 μm in diameter, composed of chlorite-group minerals. These were accompanied by weathering-related phases such as haematite and goethite on the surface of fossils in amber, and were confirmed by Raman spectra^68^. In contrast, the phyllosilicates identified in this study are clay-sized or exhibit fine sheet-like structures. Xing et al., (2016)^86^ reported voids in the exposed feather rachis/ramus (and adjacent tissue spaces) were later infilled by clay minerals, inferred from limited SEM-EDS data, which may have contributed to preserving feather morphology. Nevertheless, together with our observations, these findings suggest that clay minerals may be involved in the preservation of fossils in Kachin amber. Similarly, these mineral infills are important because their high X-ray contrast may make them among the most readily reconstructed and segmented features in micro-CT data; these phases can also stabilize morphology, thereby strongly influencing which key anatomical details of Kachin amber inclusions are most readily resolved by micro-CT.

The Fe-rich hydrous aluminium phyllosilicates, most likely chamosite or berthierine, which are commonly associated with low-grade metamorphism and low-temperature hydrothermal alteration^86^. Since amber is not expected to host such minerals, their occurrence likely results from formation during burial and diagenetic processes, either through the replacement of earlier mineral phases or by direct authigenic precipitation during burial. Therefore, these minerals in Kachin amber may have formed either (i) by direct precipitation from Fe-Mg-rich, siliceous hydrothermal fluids, (ii) through alteration of pre-existing minerals such as albite, kaolinite, and illite, or (iii) via in-situ alteration of primary volcanic fragments^87–89^. In the specimen of *H. cf. elongatus*, quartz is visibly precipitated around the clay minerals, which may be attributed to formation processes that release excess silica^87,89^. In the specimen of *C. dongi* sp. nov., the association of fine quartz crystals with clay-sized aggregates of Fe-rich hydrous aluminium phyllosilicates may indicate that they inhibited the growth of quartz cement^87^. Potassium (K) detected by EDS in the mineral aggregates was likely derived from the alteration of K-bearing minerals such as mica and K-feldspar. Their presence may indicate not only the occurrence of chamosite/berthierine but also the possible presence of other associated clay minerals. Kachin amber occurs in close association with volcaniclastic rocks^90,91^ and was preserved in a shallow, nearshore depositional environment^91^. The surrounding host rocks contain abundant lithic clasts including plagioclase, volcanic glass matrix, and quartz, as well as clay-rich material, which further supports the above inference^90^. The occurrence of clay minerals alongside other mineral phases provides additional evidence for multistage diagenetic processes, reflecting dynamic fluctuations in microenvironmental geochemistry that influenced the preservation of amber inclusions.

In our study, we further demonstrate and emphasize that minerals play an important role in the preservation of fossils in Kachin amber. Our results support a taphonomic model involving a sequential process comprising early mineralization (e.g., calcite precipitation), intermediate-stage dissolution and silicification, and subsequent precipitation of clay minerals, accompanied by microbially mediated pyrite formation. Considering the mineral assemblage associated with our mayfly fossils, including abundant pyrite, together with the mineral phases reported by Jiang et al. (2022)^68^, it is noteworthy that haematite and goethite, minerals that typically form under oxidative conditions, have also been identified. We suggest that mineralization products associated with void infills in Kachin amber may serve as useful proxies for reconstructing evolving fluid compositions, fluctuating redox conditions, and dynamic pore-space transformations within the amber and its surrounding microenvironment. Further geochemical and experimental work will be important for refining our understanding of these preservation pathways.

## Conclusion

In this study, we describe a new genus and species, *Crehkahtihtengia dongi* gen. et sp. nov., a new species, *Hexameropsis fehrmannorum* sp. nov., and a male imago tentatively attributed to *Hexameropsis cf. elongatus*, all from mid-Cretaceous amber of northern Myanmar and assigned to the extinct Mesozoic mayfly family Hexagenitidae. As with other mayflies described from Kachin amber, Hexagenitidae were likely components of the rhithral, running waters within the Burma Terrane, in contrast to the predominantly lacustrine hexagenitids known as compression fossils in sedimentary rocks of the Jurassic and Cretaceous. These new fossils expand the documented Mesozoic diversity of Ephemeroptera and enhance our understanding of the morphological variability and ecological adaptation within Hexagenitidae. Furthermore, our taphonomic analyses of *C. dongi* sp. nov. and *H. cf. elongatus* reveal the complex interactions that yielded clay minerals involved in the preservation of structures of insects in amber. The co-occurrence of calcite, quartz, pyrite, and clay minerals within single individuals indicates a complex, multi-phase mineralization history linked to microbial activity and geochemical processes. Together with previous taphonomic work on soft-bodied biological inclusions in amber and the reported co-occurrence of haematite and goethite, these results highlight the potential of amber fossils as archives for reconstructing redox dynamics, fluid evolution, and microenvironmental changes during fossilization.

## Materials and methods

The amber specimens, including three new adult mayfly fossils described here, originate from the Hukawng Valley in Tanaing Township, Myitkyina District of Kachin State, in Myanmar. The geographical coordinates of the site are approximately 26°15′N, 96°34′E. All specimens examined in this study, including *Crehkahtihtengia dongi* gen. et sp. nov. (NIGP207447), *Hexameropsis fehrmannorum* sp. nov. (NIGP207448), and *Hexameropsis cf. elongatus* (NIGP207449), are deposited at the Nanjing Institute of Geology and Palaeontology, Chinese Academy of Sciences (NIGPAS), Nanjing, China. The maximum age of Hukawng amber is 98.8 ± 0.6 Ma, based on U-Pb dating of zircons from the volcanoclastic matrix^90^. The amber pieces were collected prior to the closure of the Kachin amber mines in November 2017. The authors declare that, to the best of their knowledge, the fossil material reported in this study was not associated with armed conflict or ethnic strife in Myanmar. All fossil material was acquired in compliance with the laws of Myanmar.

Reflected light micrographs were acquired using a Zeiss AXIO ZoomV16 stereo microscope system in the Bonn Institute of Organismic Biology (BIOB), Section Paleontology, University of Bonn. Fluorescence microscopy was conducted at the Senckenberg Forschungsstation Grube Messel. Each image represents a digitally stacked composite of ~ 15–50 images captured at successive focal planes and combined using Helicon Focus 7. Line drawings were drafted using CorelDRAW Graphics Suite 2021. Figure plates were prepared using Adobe Photoshop and CorelDRAW Graphics Suite 2021.

Micro-CT data for NIGP207447 and NIGP207449 were acquired using a Phoenix Vǀtomeǀx S microCT at the Institute of Geosciences, University of Bonn. Scans were collected by a CCD-based 20x objective at 80 kV and 80uA with voxel sizes 8.91 μm (NIGP207447) and 7.57 μm (NIGP207449). To improve the signal-to-noise ratio, each specimen was scanned over 360°, with 1437 and 1412 projections acquired for NIGP207447 and NIGP207449, respectively. Volume data were processed in Avizo 8.1 (FEI Visualization Sciences Group).

Raman spectra were obtained using a Horiba Scientific LabRAM HR800 confocal Raman spectrometer at the Institute of Geosciences, University of Bonn, with excitation provided by a monochromatic laser beam with a wavelength of 532 nm.

Backscattered electron scanning electron microscopy (BSE) images of two fossil samples (NIGP207447, NIGP207449) were obtained using two SEM systems at Bonn Institute of Organismic Biology (BIOB), University of Bonn: a TESCAN VAGA 4 with (tungsten filament) equipped with TESCAN Essence™ energy-dispersive X-ray spectroscopy (EDS), and at Institute of Geosciences, University of Bonn: a Jeol 8200 Superprobe electron microprobe (EPMA) with 5 wavelength dispersive spectrometers (WDX). Samples were carbon-coated using a Quorum Q150T ES sputtering coater (Quorum Inc., Lewes, UK) before SEM-EDS or EPMA analysis. WDX intensity maps were acquired for selected elements, including Si, Al, Ca, S and Fe, using a focused electron beam, step size of 1.5 μm and a dwell time of 5000 ms.

Anatomical terminology is based on Kluge (2004)^27^ and Bauernfeind and Soldán (2012)^69^.

## Data Availability

All data generated or analysed during this study are included in this published article, or their sources are cited therein. The micro-CT data generated during the current study are available in Zenodo at [https://doi.org/10.5281/zenodo.19159009].
